# Fracture de l’épicondyle médial et latéral associée a une luxation du coude chez l’enfant (à propos d’un cas)

**DOI:** 10.11604/pamj.2018.30.87.14722

**Published:** 2018-05-30

**Authors:** Sara Hachri, Hind Abouljaoud, Hind Cherrabi, Karima Atarraf, Lamiae Chater, My Abderrahmane Afifi

**Affiliations:** 1 Service de Traumato-orthopédie Pédiatrique, CHU Hassan II, Fès, Maroc

**Keywords:** Luxation, coude, fracture, épicondyle médial, épicondyle latéral

## Abstract

La luxation du coude est une lésion relativement rare chez l’enfant. Elle représente 3 a 6% des traumatismes du coude, On constate souvent des fractures associées à cette luxation, ce sont le plus souvent des fractures de l’épicondyle médial, l’association d’une fracture de l’épicondyle latéral et médial à la fois à une luxation reste exceptionnelle. Nous rapportons le cas d’un enfant âgé de 13 ans victime d’un traumatisme du coude gauche par un mécanisme indirect. L’examen locomoteur a objectivé un coude gauche tuméfié, déformé avec une impotence fonctionnelle totale. La radiographie a mis en évidence une luxation posteroexterne du coude associée à une fracture de l’épicondyle latéral et de l’épicondyle médial qui se trouve incarcéré en intra articulaire. La prise en charge a consisté en une réduction de la luxation sous anesthésie, avec un contrôle scopique objectivant une bonne réduction, une fracture de l’épicondyle latéral jugée stade 1 selon la classification de Lagrange et Rigault après réduction et une fracture de l’épicondyle médial stade 2 selon la classification de Watson-Jones, d’où la décision d’opérer cette dernière, avec un abord postéromédial du coude. Le contrôle radiologique post opératoire immédiat et à distance jugé satisfaisant avec une ablation de l’attelle faite à 3 semaines, et une ablation de matériel d’ostéosynthèse faite à 6 semaines, une rééducation du coude était prescrite, avec un bon résultat clinique.

## Introduction

La luxation du coude est une lésion relativement rare chez l’enfant, elle représente 3 à 6% des traumatismes du coude, souvent associée à des fractures dont les plus fréquentes sont celles de l’épicondyle médial, l’association d’une fracture de l’épicondyle latéral et médial à la fois à une luxation reste exceptionnelle.

## Patient et observation

**Figure 1:**
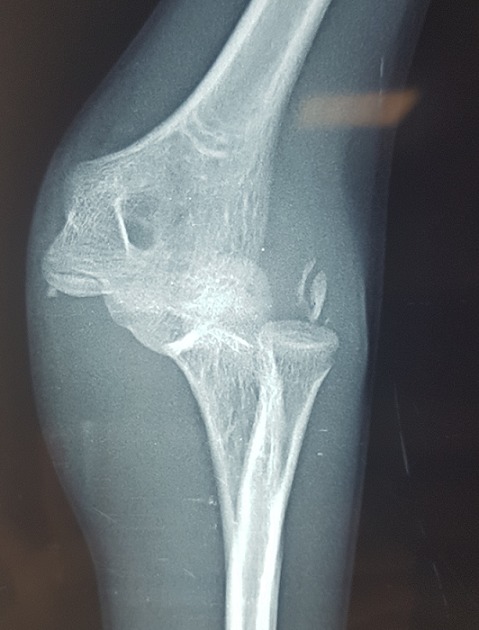
Luxation du coude associée à une fracture de l’épicondyle médial et latéral sur une radiographie de face

**Figure 2:**
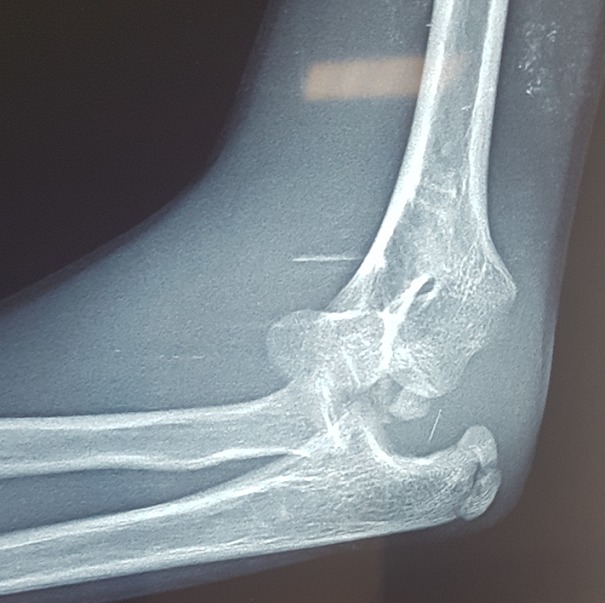
Image d’incarcération de l’épicondyle médial sur la radiographie de profil

**Figure 3:**
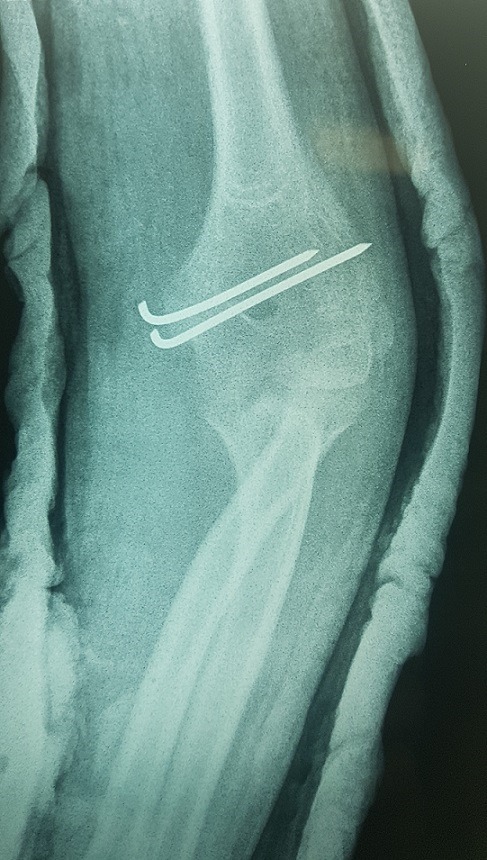
Radiographie de contrôle face en post opératoire immédiat objectivant une bonne reduction

**Figure 4:**
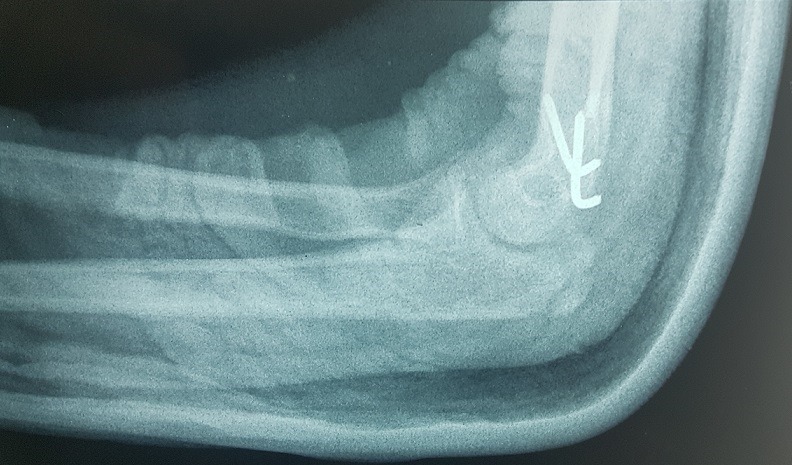
Contrôle radiographique de profil en post opératoire objectivant une bonne reduction

**Figure 5:**
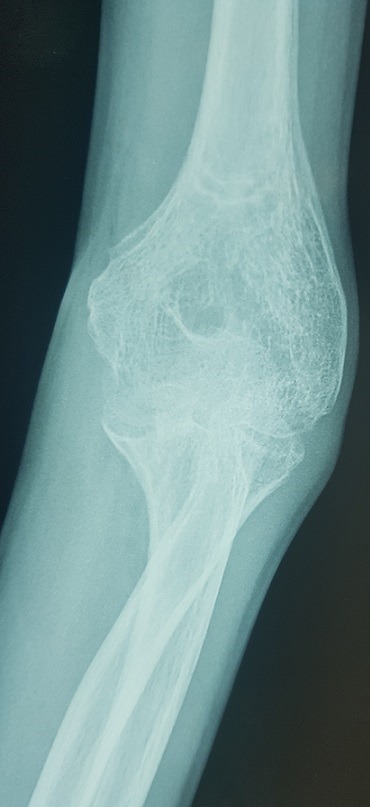
Radiographie de contrôle du coude après ablation du matériel d’ostéosynthèse montrant une bonne consolidation des fractures de l’épicondyle médial et lateral

**Figure 6:**
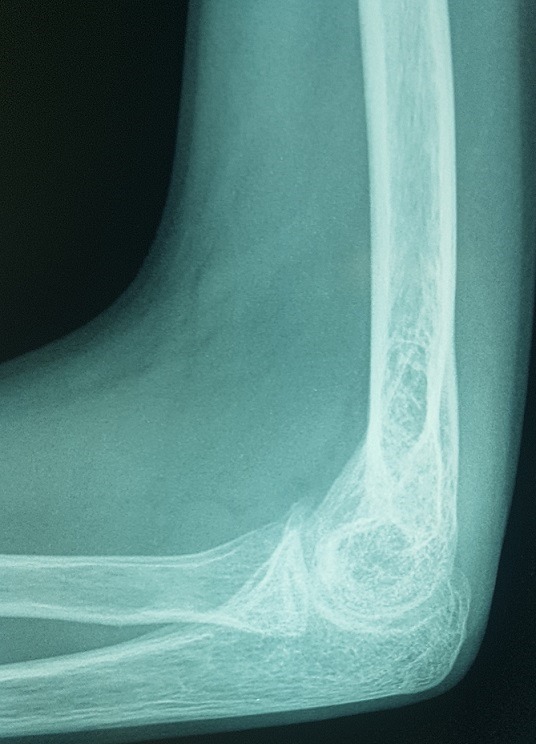
Radiographie de profil après ablation de matériel d’ostéosynthèse objectivant une bonne congruence articulaire

Il s’agit d’un enfant âgé de 13 ans victime d’un traumatisme du coude gauche suite à une chute de sa hauteur, membre supérieur en extension. L’examen locomoteur a objectivé un coude gauche tuméfié, déformé avec une impotence fonctionnelle totale, l’examen vasculo-nerveux étant sans particularité. La radiographie du coude a mis en évidence une luxation postéro externe associée à une fracture de l’épicondyle latéral et médial qui se trouve incarcéré en intra articulaire (Figure 1, Figure 2). L’enfant admis au bloc pour réduction sous anesthésie, avec un contrôle scopique per opératoire objectivant une bonne réduction de la luxation, une fracture de l’épicondyle latéral jugée stade 1 après réduction et une fracture de l’épicondyle médial qui est restée déplacée, justifiant un traitement conservateur pour la première et un abord chirurgical postéro médial pour la deuxième. Le contrôle radiologique post opératoire immédiat (Figure 3, Figure 4) puis à une semaine, à 3 semaines, et 6 semaines jugés satisfaisants avec une ablation de l’attelle faite à 3 semaines, et une ablation de matériel d’ostéosynthèse faite à 6 semaines, une rééducation du coude précoce débutée. Avec un recul de 01 an et demi, le résultat clinique et radiologique est jugé très satisfaisant, une extension complète du coude, et une flexion à 160° (Figure 5, Figure 6).

## Discussion

La luxation du coude est une lésion rare chez l’enfant, isolée elle représente 3 à 6% des traumatismes du coude, elle touche l’enfant entre l’âge de10-15 ans [1-3], le plus souvent de sexe masculin et reste rare moins de l’âge de 3 ans, on constate souvent des fractures associées à cette luxation, ce sont le plus souvent des fractures de l’épicondyle médial qui s’incarcère généralement en intra articulaire, puis les fractures de la tête radiale, l’olécrane [4] et l’apophyse coronoïde [5] , la combinaison d’une fracture de l’épicondyle médial et latéral à une luxation du coude reste une entité exceptionnelle, aucun cas pareil n’est rapporté dans la littérature. Cette lésion reste complexe du point de vue anatomique vue que l’articulation du coude aves ses différents noyaux cartilagineux qui apparaissent différemment avec l’âge peut faire l’objet d’une impasse diagnostique en dehors d’une bonne interprétation radiologique [1] et peut laisser des séquelles fonctionnelles en cas de retard de prise en charge [1]. Dans notre cas, la radiographie standard nous a permi de faire le diagnostic dans les plus brefs délais, le traitement en général d’une luxation est orthopédique consistant en une réduction sous anesthésie [6] et une réévaluation des autres fractures associées sous contrôle scopique, une réduction chirurgicale s’impose avec un embrochage par deux broches parallèles en cas de fragment déplacé de l’épicondyle médial ou latéral [5,6], une contention plâtrée est nécessaire, l’ablation se fait à la 3^ème^ semaine concomitante à une rééducation précoce [5], l’ablation de matériel d’ostéosynthèse se fait vers la 6ème semaine après consolidation et une surveillance clinique et radiologique reste obligatoire pour détecter une complication probable en cas d’ostéosynthèse associée, ou un déplacement secondaire en cas de fracture associée traitée orthopédiquement.

## Conclusion

La luxation du coude associée à une fracture de l’épicondyle médial et latéral à la fois reste une lésion exceptionnelle chez l’enfant et constitue une atteinte complexe du coude dont la prise en charge diagnostique et thérapeutique doit être précoce et adéquate pour éviter d’éventuelles complications ultérieures secondaires au développement d'un cal vicieux, ou d'anomalies de croissance par atteinte du cartilage de croissance.

## Conflits d’intérêts

Les auteurs ne déclarent aucun conflit d’intérêts.
